# Providing person-centered palliative care in conflict-affected populations in the Middle East: What matters to patients with advanced cancer and families including refugees?

**DOI:** 10.3389/fonc.2023.1097471

**Published:** 2023-03-27

**Authors:** Ping Guo, Sawsan Alajarmeh, Ghadeer Alarjeh, Waleed Alrjoub, Ayman Al-Essa, Lana Abusalem, Alessandra Giusti, Asem H. Mansour, Richard Sullivan, Omar Shamieh, Richard Harding

**Affiliations:** ^1^School of Nursing and Midwifery, Institute of Clinical Sciences, College of Medical and Dental Sciences, University of Birmingham, Birmingham, United Kingdom; ^2^Florence Nightingale Faculty of Nursing, Midwifery and Palliative Care, Cicely Saunders Institute, King’s College London, London, United Kingdom; ^3^Center for Palliative & Cancer Care in Conflict (CPCCC), King Hussein Cancer Center (KHCC), Amman, Jordan; ^4^Chief Executive Office, King Hussein Cancer Center (KHCC), Amman, Jordan; ^5^Institute of Cancer Policy, King’s College London, London, United Kingdom; ^6^Department of Palliative Care, King Hussein Cancer Center (KHCC), Amman, Jordan; ^7^Faculty of Medicine, the University of Jordan, Amman, Jordan

**Keywords:** palliative care, needs, experiences, oncology, Jordan, qualitative, refugee “crisis”

## Abstract

**Introduction:**

Universal health coverage highlights palliative care as an essential component of health services. However, it is unclear what constitutes person-centered care in populations affected by conflict, as they may have specific concerns in the dimensions of physical, emotional, social, and spiritual wellbeing. This study aimed to identify what matters to patients with advanced cancer and family caregivers in Jordan including refugees, to inform appropriate person-centered assessment and palliative care in conflict-affected populations.

**Methods:**

Cross-sectional face-to-face, semi-structured interviews were conducted at two sites in Amman. Adult patients with advanced cancer and family caregivers were purposively sampled to maximize diversity and representation. Interviews were digitally audio recorded, anonymized, and transcribed verbatim for thematic analysis.

**Findings:**

Four themes were generated from 50 patients (22 refugees; 28 Jordanians) and 20 caregivers (7 refugees; 13 Jordanians) (1). Information, communication, and decision-making. Truth-telling and full disclosure from clinicians was valued, and participants expressed concerns that information was not shared in case patients would disengage with treatment. (2) Priorities and concerns for care and support. Participants’ top priority remained cure and recovery (which was viewed as possible). Other priorities included returning to their “normal” life and their “own” country, and to continue contributing to their family. (3) Role of spirituality and Islam. Most participants had strong faith in God and felt that having faith could comfort them. For refugees whose social network was fractured due to being away from home country, prayer and Quran reading became particularly important. (4) Unmet support needs of family caregivers. Family caregivers were affected physically and emotionally by worrying about and caring for the patients. They needed support and training, but often could not access this.

**Discussion:**

Truth-telling is highly valued and essential to achieving person-centered care and informed decision-making. This study also reveals specific concerns in conflict-affected populations, reflecting the experience of prior losses and fracturing of existing social networks and support. The role of religion is crucial in supporting refugee communities, and consideration should be paid to the needs of patients and caregivers when caring for a patient at home without access to their communities of origin and the support they accessed.

## 1 Introduction

By 2060, there will be an estimated 16.3 million people experiencing serious health-related suffering and dying with cancer annually, and this number will rise more quickly in low-income countries (407% increase), lower-middle income countries (169% increase), and upper-middle income countries (96% increase), compared to high income countries (39% increase) from 2016 to 2060 (1, 2). Jordan faces unique and complex challenges in health care due to refugee influx and rising cases of cancer. It has the second-highest ratio of refugees to citizens of any country in the world, creating an immensely complicated refugee crisis (3). In Jordan, cancer is the second leading cause of death (4). According to the most recent data from the Jordan Cancer Registry, 8152 cancer cases were reported for the year 2016, of which 5999 cases (73.6%) were from Jordanians and 2153 (26.4%) from non-Jordanians including refugees. Out of the 19,676 recorded mortalities, 3084 (16.2%) were due to cancer (5). Cancer burden in Jordan is a challenge, and the national cancer control should place a high priority on developing an endorsed National Cancer Control Strategic Plan (6).

Palliative care is a multidisciplinary approach aimed at improving pain and other symptoms and optimizing quality of life among people with life-threatening illness. It is a global human right (7, 8) as well as being a powerful adjunct to oncology that adds distinct value to the physical, emotional, social, and spiritual well-being of patients living with cancer (9). Integrating palliative care into the trajectory of standard oncologic care early has shown clinical effects on alleviating symptom burden, enhancing understanding of illness and prognoses, and improving both quality of life and overall survival for patients (10). Palliative care should be provided as a crucial health service, according to universal health coverage (11). It was explicitly recognized as one of the comprehensive services needed for non-communicable diseases in the WHO global action plan for the prevention and control of non-communicable diseases 2013−2020  (12, 13). However, palliative care and pain management have received the least attention in the global health agenda. The goals of the Lancet Commission report on palliative care and pain relief can be achieved by identifying and managing the specific needs, pain and other symptoms, and concerns of patients with advanced cancer including refugees (14).

A population-based analysis of adult death registry data from the Jordanian Ministry of Health shows that the annual mortality rate rose from 6792 in 2005 to 17,018 in 2016 (151% increase). In Jordan, hospitals accounted for 93.7% of all fatalities, while both Jordanians and non-Jordanians had a rise in hospital deaths from 2005 to 2016 (82.6%–98.8% and 88.1%–98.7%, respectively) (15). The need of palliative care is rapidly increasing; however, palliative care is not routinely available across Jordan (4, 16). In addition, Jordan is experiencing difficult healthcare issues as a result of the influx of refugees from numerous regional conflicts, notably Syria. The lowest expenditure of cancer care for Syrian refugees is estimated approximately 2.09 million euros annually (17). Using diagnosis-specific cost information from the King Hussein Cancer Foundation, it is projected that 869 Syrians are diagnosed with cancer each year in Jordan, costing roughly 15.6 million Jordanian dinars (US$22.1 million) for their treatments (18).

Access to cancer treatment is poorer for refugees and immigrants than for other Middle Eastern citizens (19). Prior to, during, and after displacement, they frequently encounter stressful and traumatic situations (20–22), as well as a heavy burden of physical, emotional, mental, and financial obstacles (23). Up until recently, humanitarian emergency and crisis did not include palliative care, which provides holistic care and support to people who are experiencing serious health-related suffering (24). What matters to patients with advanced cancer and their caregivers from refugee population in Jordan has not been explored. A systematic review indicated a lack of evidence on palliative care needs and interventions in humanitarian crises, difficulties in providing palliative care particularly in the context of limited resources and guidelines, and the importance of contextually appropriate care (25). The COVID epidemic revealed both potential and deficiencies in the palliative care response to health system need to provide better care at the end of life (26). In addition, it is unclear what constitutes person-centered care in populations affected by conflict, as they may have specific concerns in the dimensions of physical, emotional, social, and spiritual wellbeing. This study aimed to identify what matters to adult patients with advanced cancer and family caregivers in Jordan including refugees, in order to inform appropriate person-centered assessment and palliative care in conflict-affected populations.

## 2 Methods

### 2.1 Study design

We explored the unique needs, specific experiences of, and preferences for palliative care from the viewpoints of patients with advanced cancer and family caregivers in Jordan using a qualitative cross-sectional design through individual interviews. The guidelines of the Consolidated Criteria for Reporting Qualitative Research (COREQ) were adhered to (27). COREQ is a 32-item checklist for explicit and comprehensive reporting of in-depth interviews and focus groups.

### 2.2 Setting and participants

The study took place at two sites in Amman, Jordan: a comprehensive cancer center that manages around 60% of the country’s cancer cases and a public hospital that offers some supportive care. The eligible participants in this study included patients who were ≥18 years old with advanced cancer and received care at one of the study sites, and informal caregivers who are defined as “unpaid, informal providers of one or more physical, social, practical, and emotional tasks. In terms of their relationship to the patient, they may be a friend, partner, ex-partner, sibling, parent, child or other blood or non-blood relative” (28). People from Jordanian and refugee populations who met the inclusion criteria were invited to participate in this study. Patients deemed too unwell to be contacted by healthcare professionals, and those unable to speak Arabic or English were excluded from the study. In order to maximize variation in age, gender, place of origin, and primary diagnosis, participants were purposively sampled. Patients and informal caregivers were recruited and interviewed separately.

Oncologists, palliative care physicians, nurses and other healthcare professionals who were working at the two sites during the study period helped to identify and first approach the eligible patients and family caregivers. They received a study training session which covered the topics of the eligibility criteria, how to identify and approach potential participants, and informed consent process. Then they applied the knowledge and skills learned from the training to screen eligible patients including both inpatients and outpatients and provide them with information sheets. The dedicated research team contacted those who expressed their interest in taking part in this study, gave them more information about this study, and responded to any question they had. Similar consent process was adopted for recruiting caregivers. 24 hours or longer were given to all potential patients and caregivers if they desired more time to consider their participation. Written consent (or an ink thumb print if unable to write due to disability, weakness, or illiteracy) was obtained from those who decided to participate. All study materials including information sheet and consent form were translated from English into Arabic using forward/backward translation to enhance accuracy.

### 2.3 Data collection

Between October 1, 2018 and May 31, 2019, the research team conducted in-depth semi-structured interviews with participants in their preferred time and locations. The interviewers (SA, GA, WA, and LA) have a wealth of knowledge and experience in both qualitative research and palliative care. Before participants gave their consent, the interviewers had no relationship with them. The development of topic guides for patients and caregivers was informed by a review of the research evidence on the experiences of refugees in Jordan (21, 22, 29, 30), and the needs of palliative care in humanitarian emergency and crisis settings (24, 25). The topic guides were then improved through regular discussion among wider cross-national research team members.

The topic guide for the patient participants covered (a) the experiences of having a terminal illness, facing death, and receiving support from family; (b) personal priorities, needs, and concerns, (c) the needs, experiences, and preferences of patients with advanced cancer in terms of palliative care (such as family needs, religious/spiritual needs, trauma/displaced experience/challenges as refugees, preferences for advance care plan, decision-making, and communication); and (d) what would make care more patient centered. Apart from these, the needs and concerns of the family caregivers themselves, as well as their experiences with providing care for a person with advanced cancer, and opinions on the quality of care were also explored. Data were collected until we reached information power (i.e., no new themes were identified in line with the study aim). Each interview was digitally audio recorded and the interviewer took field notes if needed.

### 2.4 Data analysis

These interviews were anonymized, transcribed verbatim by GA, and then translated from Arabic into English by AA. The researcher from the United Kingdom (UK) (PG) and the researcher from Jordan (SA) jointly coded the data in QSR NVivo 10, which were then analyzed using thematic analysis. To establish a coding framework, the two researchers independently examined seven transcripts. They then used that framework to analyze the remaining transcripts. As the data collection process continued, the data were simultaneously transcribed, translated, and analyzed. The categorization and comparison of interview data allowed for the discovery of recurrent themes and sub-themes in the dataset. Themes that overlapped were combined under descriptive names, while themes with few quotations were investigated. The data were continually examined during this process to learn more about the connections and relationships between the themes. Researchers discussed the themes and sub-themes when they were emerging to increase reliability and dependability.

### 2.5 Ethics and project management

The Research Ethics Committee at King’s College London (HR-17/18-7243), King Hussein Cancer Center (18KHCC70), and the Jordanian Ministry of Health (MOH REC 1800110) granted relevant ethical approvals. The study complied with the Declaration of Helsinki (31). Throughout the study, the research team upheld the study’s integrity, patient privacy, and data confidentiality. The UK and Jordanian members of the steering committee regularly met to discuss the study’s overall direction and review its progress.

## 3 Findings

### 3.1 Participant characteristics

A total of 71 eligible people were approached and invited to participate in the study, of which only a native Jordanian declined. No difference was identified in the overall response rate or willingness to participate in the study between native people and refugees. 70 participants (50 patients with advanced cancer, 20 family caregivers) were interviewed including 29 refugees (22 patients, 7 caregivers) (**Table 1**). Patients ranged in age from 26 to 75, with an average age of 54 years. They were of six nationalities (Iraqi, Jordanian, Libyan, Palestinian, Syrian, Yemeni). Refugees’ duration of stay in Jordan ranged from 6 months to 50 years. All the participants were Muslim except one patient who was Christian. The length of interviews varied from 12 to 97 min (Mean=60). All participants answered the questions in the topic guide. Only in two interviews conducted with the patients, their family caregivers also joined the interviews to accompany the patients but made no or limited contribution to the actual discussion.

**Table 1:** Participant characteristics (n=70).

	Patients (n=50)	Caregivers (n=20)
Mean age in years (range)	54 (26-75)	42 (19-67)
Gender		
Male	20	7
Female	30	13
Primary diagnosis of the patients		
Breast cancer	20	5
Colorectal cancer	9	5
Lung cancer	5	3
Gastric cancer	3	1
Lymphoma	2	0
Gallbladder cancer	1	1
Bladder cancer	1	1
Pancreatic cancer	1	0
Parotid gland adenoid cystic carcinoma	1	0
Prostate cancer	1	0
Myelofibrosis	1	0
Ovarian cancer	1	0
Rectal cancer	1	0
Skin cancer	1	0
Anal cancer	1	0
Acute lymphocytic leukaemia (ALL)	1	0
Brain tumour	0	1
Liposarcoma cancer	0	1
Orbital tumour	0	1
Sarcoma cancer	0	1
Cancer stage of the patients		
III	13	2
IV	36	18
Unstageable	1	0
Number of people to live with		
Live alone	3	0
1-5	36	12
6-10	10	2
11-15	0	1
16-20	0	1
Unknown	1	4
Care for a sick family member?		
Yes	7	3
No	1	0
Unknown	42	17
Number of children < 18 years of age		
None	29	8
1-3	16	7
4-8	4	2
Unknown	1	3
Nationality		
Jordanian	28	13
Syrian	11	3
Iraqi	4	0
Libyan	4	1
Palestinian	2	2
Yemeni	1	1
Refugees’ duration of stay in Jordan		
≤2 years	7	2
3-5 years	2	1
6-7 years	8	1
≥8 years	2	0
Unknown	3	3

### 3.2 Themes

Four main themes were identified (1): Information, communication, and decision-making; (2) Priorities and concerns for care and support; (3) Role of spirituality and Islam; and (4) Unmet support needs of family caregivers. These four main themes and ten sub-themes are presented in **Table 2** and supported by direct quotations from the interview participants below. A unique combination of three letters and four numbers was used to represent each participant (such as P or C indicating the type of participants - patient or caregiver, followed by KH or AL indicating the study site and then four digits indicating participant numbers).

**Table 2:** Themes and sub-themes.

Themes	Sub-themes
Information, communication, and decision-making	Information needs Honest communication Informed decision-making
Priorities and concerns for care and support	Getting back to normal and managing pain Social support and financial challenges Being able to contribute to their family and society
Role of spirituality and Islam	Strong faith in God Believing in God or taking medications
Unmet support needs of family caregivers	Caregiver burden Support and training needs

#### 3.2.1 Theme 1: Information, communication, and decision-making

***Information needs***

Most participants believed that the patients had the right to know everything about their condition.

“I want to know which stage the disease have reached. Is it bigger? Is it smaller? Maybe it’s gone! But they just prescribe me meds and painkillers.” (PKH0014, Jordanian)

The patients emphasised the importance of face-to-face consultations and valued hearing the opinions of more than one medical doctor. Mutual trust was described as a fundamental aspect of the doctor-patient relationship. Building strong trust-based relationships with the patients could help to achieve their optimal health outcomes and care experiences.

“I prefer coming in person. I am already coming here so I felt that it is better to consult them face to face. Patients’ lives are precious, so there should be more than one opinion involved. A doctor can make mistakes, but when it is more than one doctor, the chance of making mistakes is smaller.” (PKH0018, Jordanian)

When the patients were informed about their condition and treatment plan, they felt that their worries and anxiety were reduced, and they had more trust in the clinicians treating them (e.g., PKH0020, refugee; CKH0008, refugee).

“If I have a question, I would ask the doctor. He’s the one responsible for my case so he would be more helpful. I think patients would prefer to have information directly from their doctor rather than online.” (PKH0024, Jordanian)

“A doctor is a person who cares about their patients and not see them as a source of money and still care because the reward of the doctor is not the money, the reward is that God would bless that money and effort. A patient life is trusted in the hands of a doctor and that’s why they should be accurate as this is a serious disease and should attend to the patient’s complaint.” (PKH0034, refugee)

Most participants expressed the view that doctors played an important role in information giving. They believed that doctors were good sources of information (but not nurses). Heads of department were seen as being more knowledgeable and respected. The health care system is reportedly embedded in a hierarchical culture and structure where the voice of doctors and senior managers is often given more importance than others. Both patients and caregivers considered that receiving positive information could boost patient spirits and make a significant difference in their physical and psychological wellbeing, even though later it was honesty that was valued.

“… he (the patient) would listen to a doctor rather than a nurse. Because he likes to hear the information from the source, so when it is from these two doctors, he listens because I think they’re the head of department. Because they told him from the beginning that everything was ok and because they gave him a morale boost. He trusts the doctor more than he trusts his son, because his son might lie to him to make him feel better and he knows that his son’s love will prevail, but the doctor will answer his questions so he will trust the doctor more.” (CAL0014, refugee)

***Honest communication***

Participants commented that lack of honest communication and disclosure was not valued and although prayer was very important, it should not be used as a euphemism for breaking bad news. They also expressed concerns that information was often not shared in case it would cause patients to disengage with treatment.

“I remember asking the nurse in Saudi Arabia about my mother’s condition and she told me there was nothing we could do but pray. She should have told me about her condition, I am her companion, I can handle it, but what she said meant she was going to die.” (CAL0016, Jordanian)

“Maybe they don’t provide adequate information because they worry about the patient’s feelings and to ensure they accept the treatment. If you tell a patient their body is no longer responding to the treatment, they might decide on pulling the plug on the whole thing.” (PKH0024, Jordanian)

However, some participants highlighted that clinicians would need to know their patients well enough to be able to assess whether their patients could accept bad news and judge when would be the appropriate time to communicate this news to their patients. Patients reported that communication must be personalized and raised the importance of clinicians correctly judging whether to communicate with patients directly or *via* family. A few participants expressed the views that clinicians should only inform the family of ‘bad news’ as a way to protect the patients.

“The doctor should also be smart enough and know that some patients cannot tolerate certain news so the doctor should have an insight. He should know I am a nosy person and wanting to know every single detail, otherwise I won’t receive treatment, whereas other patients cannot tolerate such news and he should inform his family instead.” (PKH0017, Jordanian)

“She should tell the family, but I am against telling the patient. He would be crushed regardless of how strong his faith is … In my opinion, patients shouldn’t be told they are terminal, or that they are not to be given chemotherapy anymore. They should leave that to God. I wished I had met his doctor, but I didn’t, I wanted to tell her she shouldn’t have told him whatever she told him.” (CKH0011, refugee)

***Informed decision-making***

Other participants also explained their preference of being informed about different treatment and care options beforehand and getting timely advice. This would help them to make informed decisions.

“They should tell him everything they intend to do and give him the chance to make decision, even regarding the type of painkiller he wishes to be on. They should tell him about possible complications. The symptoms I experienced from the chemo really burdened me. I didn’t know anything when I took the first cycle, so I advise anyone who is sick to tell their doctors to explain to them before taking any step.” (PKH0027, Jordanian)

In addition, timely access to information and internal team communication was crucial to patients and they valued this as a marker of quality care.

“We have seen the palliative doctors three or four times since the admission. They come and talk to him (the patient) then they talk to us outside. If it happens that I don’t catch the palliative doctor I can always go see him on the second floor and ask him … They are great when it comes to follow-up. Even the nurses. They have good communication between each other and if anything comes up they immediately contact the patient…” (CKH0007, refugee).

#### 3.2.2 Theme 2: Priorities and concerns for care and support

***Getting back to normal and managing pain***

Among our sample of patients with advanced disease, their priorities included cure and recovery (which was viewed as possible), returning to their “normal” life, and to continue contributing to their family (e.g., CAL0001, Jordanian).

“I wish I could go back to my normal life … I want to be active again. I can beat the disease, I just need to feel better and at ease, so I can beat this disease faster.” (PAL0040, refugee)

“I have a family that I am responsible for, I might be an old man, but I still care about them and I still hold myself accountable for them. This responsibility makes me worried, of course it does, whom would I worry about if it wasn’t my kids? Myself?” (PAL0045, refugee)

Some participants stated that managing pain was a top priority for them. Family was still often a dominant theme when discussing pain.

“I want my pain to calm down so that I can be there for them … I want to reduce the pain so I can be around them again. Go out with them on the weekends.” (PKH0011, Jordanian)

***Social support and financial challenges***

For all refugees, they expressed the view that they would like to receive cancer treatment in Jordan and hoped they could return to their home country eventually.

“We have two homelands; one that lives in us, and another in which we live in. Jordan is our home which we live in, respect, and appreciate. We are ready to fight for it. As for our home that lives in us, we hope one day we will return to it. The disease made me stronger. I don’t think about my disease. I believe that this illness doesn’t make my life any shorter … So my lifespan has nothing to do with this disease.” (PAL0010, Jordanian)

Some refugees raised concerns that they could not afford medication and treatment any longer and that they were experiencing financial challenges. Any financial aids that could help them to get medication and cover basic living expenses would be a priority.

“There isn’t any money. I wish they would help us with the pain and lower the price of the medication.” (PAL0002, refugee)

“I worried about my kids. Financially, I want them to be covered, but my financial status is below zero. We rented two rooms in a house that leaks when it rains and has no heating.” (PAL0037, refugee)

“Its life and the hardships we’re facing. I am burdened financially and mentally. I cry constantly because our life is sad, and we suffer financially. It is the money, the loss of my daughter and my illness. Yes, because we don’t have any money.” (PAL0009, refugee)

***Being able to contribute to their family and society***

A few participants mentioned that they did not worry about their own disease but were more concerned about their family and other people. Family roles and responsibility mattered most to many participants. They had a sense of needing to put things “in order” and plan ahead. This links strongly to the theme about honest communication about their conditions.

“I am not worried about the disease, but I want to make sure my kids have a good future. My son is 26, if he had gotten married, wouldn’t I be seeing his kids already? Seven years ago, I thought I was going to die because I had cancer, so I wed my other son, he was 22 years old at the time.” (PAL0044, Jordanian)

Furthermore, their priority was to be able to continue to help others and contribute to society.

“When I was going to surgery, the doctor asked me if I was afraid of anything, and I told him “What would I be afraid of? The Prophet said my people live sixty to seventy years, and I have already passed the limit by 5 years”. He started laughing. Why would I be scared? I see this disease as a blessing, not a curse … I help people. There is nothing better than helping people. When you help people, you feel that you exist. This is a person’s value in the society. You can’t put a price on helping people out. In the association, we help out orphans and people in need.” (PAL0010, Jordanian)

#### 3.2.3 Theme 3: Role of spirituality and Islam

***Strong faith in God***

Most participants expressed their strong faith in God and felt that having faith comforted them and helped to prevent worries, anxiety, and depression (e.g., PAL0002, refugee; PKH0032, refugee; PAL0041, refugee; PAL0037, refugee; CAL0013, refugee).

“I feel relieved when I pray, it’s like my burden were taken away. I love praying … I have more faith and trust in God because he is with me and he answered my prayer, he never forgot about me. It makes me strong, and my faith grows stronger, it gives me strength … If I let desperation into my life, I will feel worse. I already have the disease so why make it harder for myself? I pray, I strengthen my faith and be optimistic. Those around me grant me strength. I am optimistic and I believe I will get out of this, and that God will cure me.” (PAL0038, refugee)

“We are all believers, but when you are in this condition, you feel like you need to stick to your prayers and all the forms of worship, I became more adherent to them. I know they all love me and pray for me, but it is God who will cure me, I always ask him to strengthen me, and that’s when I feel that he loves me, I like to think that he is putting me through this as a form or redemption from my sins or to get me closer to him…” (PAL0040, refugee)

“I pray, praise be to God. I always read the Quran and rarely skip a prayer, I do it as much as I can. I pray in the mosque and go to Jumaa Prayers [Jumaa Prayer is a special prayer held at noon time every Friday, it includes a short lecture and a prayer. It is similar to Sunday services in Christianity].” (PAL0006, refugee)

***Believing in God or taking medications***

Furthermore, participants believed that a cure for their illness would be in the hands of God, and that they could leave matters to God and God’s mercy would exceed all, even though they also valued the use of medications.

“I am not concerned about the progression of the disease because I have faith in God. Sometimes I worry that my pain would be unmanageable, but I think increasing the morphine would help with that. Like I said before, I am content with whatever God has written for me. Life and death are a godly matter…” (PAL0007, refugee)

“I talked to him (my son) over the phone, and I started to read some Quran to him when I came here and he felt better … I believe in what God said in the Quran; “And we send down of the Qur’an that which is a cure and a mercy to the believers”. It is all about believing. If you believe the Quran will cure you, then it will. God grants us immunity when we believe in him, and if this immunity is disrupted, all the medications in the world will not help … We do what we can do and leave the rest to God. We try to make all causes come and leave the rest up to God. We knock every single door, believing it will eventually come up to God … I don’t believe it is serious because I have faith in God that there isn’t a disease without a cure. It could be something very simple. We just have to take every measure and pray to God to cure him. I know a stage 4 cancer is serious, but I know God’s mercy exceeds all.” (CAL0013, refugee)

Participants highlighted that their social network was fractured due to being away from their home country, and prayer and Quran reading became the most comforting and meaning thing they could do.

“God is all I have. What can I possibly do? I go out and sit by myself. I cry a little, then I leave things in the hands of God. If was in Syria, I would be surrounded by my family and relatives, but here I have no one. He (my son in law) reads from the Quran and prays. Some people started praying or reading Quran after him getting diagnosed, but nothing changed for him because he already did that even before his diagnosis. These last days he likes to read from the Quran when he’s alone.” (CKH0007, refugee).

#### 3.2.4 Theme 4: Unmet support needs of family caregivers

***Caregiver burden***

Participants described family members having *“their world turned upside down”* (PKH0020, refugee). They worried about the patients, became sleepless, and reduced the time they spent away from home and stopped socializing with others (e.g., CKH0008, refugee). Patients were aware that their family members knew more about their conditions than them, which reconfirmed that families were often informed instead of the patients. This brought a great burden to family caregivers.

“They (family caregivers) know I am sick, and they know I have the disease. They know more than I do because they ask the doctors. They worry about me. They worry I might feel sad or anything. They are more informed about my illness … my disease affected the family…” (PAL0037, refugee)

Family caregivers often lost social and working life due to their caring role and had to manage the mental health of the patient. They also often suffered from their own poor health.

“I used to be out of the house most of the time, I would stay at home for a maximum of one to two weeks, but since my mother got sick, I am staying with her. It all depends on how well you accept the fact that this is fate, and you can’t change it. It is tough for her, she thinks anything told to her is because she is dying, she becomes very sensitive and angry in an intolerable way.” (CAL0016, Jordanian)

“I stopped going out after he got sick. I don’t visit people anymore … There is nothing else I can do. I can’t physically take care of him, I am an old lady and I have diabetes.” (CAL0004, Jordanian)

***Support and training needs***

Family caregivers felt sad when they saw how the disease had changed their loved ones but there was nothing they could do to help. When they received bad news, family caregivers had to conceal or give a positive interpretation of poor prognosis, and this brought a psychological burden on the family.

“There was nothing I could do. He’s a 23-year-old boy and my daughter’s fiancé, surely that would make me upset. I felt sorry for my daughter who comes with him every day. I didn’t tell her that the cancer had spread in his entire spine … I am trying to spread some positivity in the air because she spends her time crying … What do I do? I talk to her and try to cheer her up. I tell her “Let’s have a cup of coffee” or “let’s go do some grocery shopping”. I try to change her mood. I’m not fine myself. It hurts me to see how he was and how he is now. I cry a lot, but not in front of him. There is nothing we can really do.” (CKH0007, refugee)

“It has a psychological impact, of course, when you see your own son not able to go to the bathroom, of course you’ll feel bad. When he is a young man at that age and to be this disabled, it kills you to see your son like that. It kills me to see my son helpless … My own son who was my rock, to see him being destroyed like this. He was really helpful to me, he carried them on his shoulder, and to see him like this. He was a pillar in this family.” (CAL0013, refugee)

They were affected physically and emotionally by worrying about and caring for the patients. Family caregivers need support and training but often could not get it. They felt guilty when they saw their relatives suffering but could not help.

Not just courses on how to deal with a sick person and talk to them, I believe these are skills that should be passed on to all people surrounding the patient because when a person gets sick, all of those around them are also sick … When you see a person going through so much pain and you can’t do anything about it, that makes you feel guilty. When you have parents who are at an advanced stage, it is nice to provide guidance to accept it and explain to them that there is nothing they can do. This is very important, because providing support to the family at such a stage is very important. (PKH0017, Jordanian)

Some young caregivers stopped education because of their caring role. Even the children in the family having patients with advanced cancer, tried to offer help beyond their age (e.g., PAL0040, refugee).

“I stopped studying. I failed high school because I only wanted to help my mother. She kept encouraging me to study and I told her I only wanted to be around her and help her.” (CAL0001, Jordanian)

This was a real challenge for young children when they were seeing very sick parents but being told they were well.

“I tell them he’s well. No, because they see him at home and how weak he is, sometimes he can’t get out of the bed or can’t eat. They cry sometimes and say “I don’t like seeing daddy like this”.” (CAL0003, Jordanian)

## 4 Discussion

This study reveals the importance of honest communication, community support and spiritual care for people with advanced cancer in Jordan and highlights the impact of the unmet needs of family caregivers. Open and effective communication about their cancer illness, treatment plan, and appropriate spiritual and psychosocial support have been identified in our study as the things that matter most to patients and family. Yet, these aspects of care are often underserved or overlooked.

Health literacy is crucial in health care, particularly in the context of cancer where patients and family caregivers experience clinical uncertainty and suffer overwhelming physical, psychological, social, and spiritual symptoms, and financial burdens (32, 33). Research shows that effective person-centered communication between health care providers and patients can improve patients’ health literacy, which also leads to enhanced health outcomes (34–36). An adequate level of health literacy is required to make informed decision, and several studies have reported that most cancer patients express a willingness to have their diagnoses and prognoses fully disclosed and show preference for participation in treatment decisions. However, according to the family caregivers interviewed in this study, patients’ knowledge of their diagnosis and prognosis could have a negative impact on how their illness progresses in the future and lead to stress, despair, hopelessness, and low self-esteem in patients. Therefore, they preferred nondisclosure of information (37, 38). These findings were corroborated in a multicentre cross-sectional qualitative study in India, which demonstrated that while clinicians were motivated by professional values to disclose cancer related information, they experienced pressure from family members to conceal information (39).

Despite the fact that the majority of patients expressed a preference of face-to-face consultations for full disclosure of their cancer diagnosis and prognosis, family members frequently make decisions in many non-Western societies (40–42) and non-disclosure is more likely to occur in “family-centric” Asian cultures (43). A systematic review of 53 studies which explored prognostic disclosure in cancer care using different communication methods and interfaces found that cancer patients usually know their diagnosis, and to some extent, their prognosis, even though their clinicians and family members do not disclose information (44). In our study, some participants expressed the views that clinicians should know their patients well to be able to judge whether to communicate with patients directly or *via* family, particularly when breaking bad news. Patients often hesitated to ask questions and were not involved in decision-making process, which potentially led to patients’ poor insight into their disease, lack of consent to treatment, and limited access to palliative care. In addition, there was a clear hierarchy within the team in terms of information giving and communication. Our findings suggest that patients strongly trusted the oncologist and people in senior leadership roles (such as Director of the Department), and doctors were seen as good sources of information instead of nurses.

How the patients and their families who were interviewed view disease, suffering, and death and dying processes is greatly influenced by spirituality and Islam (45, 46). According to a systematic review, patients and their families accepted their diagnoses because they believed that Allah (God) planned their destiny and path. They developed coping mechanisms by engaging in Islamic practices such as praying to Allah and reading the Qur’an (the holy book of Islam) which brought them hope, internal peace and comfort (47). It is reconfirmed by our findings in this study indicating that patients and family caregivers became closer to God and performed prayers particularly in the context of advanced cancer and when they were experiencing pain. They believed that Allah has more power than medication and could give them the strength to conquer everything.

Some of the participants in this study are refugees (n=29) who experienced compounding trauma related to war and disease and were receiving cancer care in Jordan. They reported that their social support network was fractured, therefore, religious practice and prayer were described as even more important for them, which has been published in a separate paper (23). A cross-sectional study with 95 palliative care outpatients has shown that spiritual wellbeing had a positive correlation with quality of life and the dimensions of physical, emotional, and functional wellbeing. In addition, it was associated with being less uncertain, more satisfied with one’s decision, and feeling more informed and supported (48). It is crucial to determine the role and influence of spirituality in palliative care, however, evidence is limited on the perceptions of spirituality among Muslim patients with advanced illness and their families.

Financial challenges were also identified as key issues refugees were experiencing in our study and they often could not afford health care costs and basic living expenses. It is recognized that migrants usually undergo a difficult integration process to live in the hosting community. This process involves the cultural and economic adaptations (49). Our findings indicated that accessing to and receiving cancer treatment and palliative care under the hosting healthcare system added another layer of complexity into the life of refugees. In addition, the relationship between migrant patients and health care providers may be impacted by cultural differences (50) and communication barriers (51). For example, clinicians may be reluctant to discuss health conditions and care with migrant patients as communication may take more time and effort. Refugees may consider that communication with their clinicians results in stereotyping, so become less likely to share their feelings, needs and concerns with them (52).

Cancer patients particularly refugees in Jordan often experience unmet multidimensional needs, symptoms, and concerns. Person-centered care is considered as an essential component of high-quality healthcare for people with palliative care needs across the globe (53). In a cross-national, cross-sectional qualitative study, interdependency and collectivism have been highlighted in relation to person-centered care, including “the benefits of social support systems, the value of interpersonal harmony, the interdependence of a person’s and their loved ones’ well-being, and the value of group-based learning, peer-to-peer support, and care in the community”. According to this study, in order to provide person-centered care, the healthcare system must have certain structural components in place such as partnerships with community-based workers and professional training in person-centered care values (54).

In this study, a lack of ability to engage in family responsibilities due to illness and treatment was identified as a priority concern of the patients. Continuing “responsibility” to family was seen as important. A few participants mentioned that their priority was to be able to continue contributing to their family and that they were more concerned about their family and other people than about their own disease. Evidence suggests that caring for a loved one who is nearing death is often physically and emotionally intensive. In addition, family caregivers of patients with advanced cancer frequently experience high level of distress, depressive, and anxiety symptoms, which highlights the critical need of psychological and emotional support for both patients and their family caregivers (55–57).

However, family caregivers’ own needs are often unidentified, unmet, and neglected. The level of care burden can be persistent or increasing over time, particularly in the care situation of refugees with advanced illness where they have two homelands, one which lives in them and another which they live in. As a result of being cut off from their wider social support network due to being abroad, caregivers experienced heightened anxiety and responsibility (23). In addition, our study reveals that children wanted to be involved but were often shielded and excluded by adults throughout parental life-limiting illness. Children’s experiences of caring for a dying parent were reported emotionally taxing and the required heavy caregiving responsibilities may have had an impact on their academic performance. This is consistent with the findings of a review ensuring that the voices of children whose parents have terminal diseases are heard and acted upon (58).

Prior studies have suggested that staff shortages and budget constraints in Jordan (especially at public hospitals) make it difficult to identify palliative needs of patients and provide holistic care (59). These findings can help improve access to palliative care by structuring assessment and services. A regular holistic needs assessment with standardized instruments such as the Integrated Palliative care Outcome Scale (IPOS) is required for early identification and intervention of symptoms and concerns (60). Adequate training including symptom management, communication, psychosocial and spiritual care is important for healthcare providers to strengthen their capacity to deliver dignified and person-centered cancer and palliative care. In addition, to make it easier for family caregivers to balance paid work with caregiving responsibilities and lower the risk of burnout during the trajectory of an illness and after death, greater support is required. Families should be trained and given the skills to care for their loved ones with advanced cancer.

## 5 Strengths and limitations

To our knowledge, this is the first qualitative study to determine what matters to patients with advanced cancer and family caregivers in Jordan including both host Jordanian and refugee populations. It not only emphasizes the importance of identifying and addressing multidimensional needs of patients and their families, but also provides recommendations of how person-centered and culturally appropriate palliative care could be better implemented to improve outcomes. We recruited the participants from two study sites – a specialist cancer center and a public hospital, enabling that highly diverse experiences and needs of people from different backgrounds have been represented and captured. Cross-national collaboration has also made it possible to acquire, analyze, and interpret data from a variety of cultural perspectives.

A study limitation is a relatively small sample size of refugee population for family caregivers (7/20, 35%). The responses to the topic guide may have been more diverse and in-depth if there had been more refugees from caregiver participants. Additionally, more patients with breast cancer and family caregivers (n=20 patients and 5 caregivers, 36%) and Syrians (n=11 patients and 3 caregivers, 20%) were interviewed than the other groups, which might have made it harder to compare and contrast the viewpoints from other groups. Future research should explore the needs and experiences of cancer and palliative care among patients with other cancer conditions than breast cancer and refugees from a variety of original countries.

What matters to people with advanced cancer and their families is unique and context specific. It is not appropriate to immediately extrapolate data generated from Western countries to Muslim-majority nations like Jordan, where 95% of the population follows Sunni Islam (61) and there is the second highest share of refugees per capita globally - most affected by the Syria crisis (3). Further investigation is needed to understand its cultural and spiritual assumptions, and the population with the underpinning evidence generated should be scrutinized.

## 6 Conclusion

Our study gathered first-hand information about the multidimensional and unique needs of patients with advanced cancer and families such as communication and information needs, and explored the role of spirituality and Islam, and the impact of unmet family support. It provides a robust evidence base to inform culturally appropriate cancer and palliative care in Jordan. Truth-telling is highly valued and essential to achieving person-centered care and informed decision-making. This study also reveals specific concerns in conflict-affected populations, reflecting the experience of prior losses and fracturing of existing social networks and support. Religion plays a crucial role in supporting refugee communities, and consideration should be paid to the needs of patients and family caregivers when caring for a patient at home without access to their communities of origin and the support they accessed.

## Data availability statement

The raw data supporting the conclusions of this article will be made available by the authors, without undue reservation.

## Ethics statement

The study involving human participants was reviewed and approved by the Research Ethics Committee at King’s College London (HR-17/18-7243), King Hussein Cancer Center (18KHCC70), and Jordanian Ministry of Health (MOH REC 1800110). The patients/participants provided their written informed consent to participate in this study.

## Author contributions

RH and PG conceptualized and designed the study, with input from SA, AM, AG, RS, and OS. SA, GA, WA, AA, and LA were involved in data collection, transcription, and translation. PG and SA jointly analyzed and interpreted the data. PG wrote the manuscript. All authors contributed to the article and approved the submitted version.
